# Three Physiological Components That Influence Regional Cerebral Tissue Oxygen Saturation

**DOI:** 10.3389/fped.2022.913223

**Published:** 2022-06-13

**Authors:** Ena Suppan, Gerhard Pichler, Corinna Binder-Heschl, Bernhard Schwaberger, Berndt Urlesberger

**Affiliations:** ^1^Division of Neonatology, Department of Pediatrics, Medical University of Graz, Graz, Austria; ^2^Research Unit for Neonatal Micro- and Macrocirculation, Medical University of Graz, Graz, Austria; ^3^Research Unit for Cerebral Development and Oximetry Research, Medical University of Graz, Graz, Austria

**Keywords:** regional cerebral tissue oxygen saturation, near-infrared spectroscopy, oxygen content, circulation, oxygen extraction, neonate

## Abstract

Near-infrared spectroscopy (NIRS) measurement of regional cerebral tissue oxygen saturation (rcStO2) has become a topic of high interest in neonatology. Multiple studies have demonstrated that rcStO2 measurements are feasible in the delivery room during immediate transition and resuscitation as well as after admission to the neonatal intensive care unit. Reference ranges for different gestational ages, modes of delivery, and devices have already been published. RcStO2 reflects a mixed tissue saturation, composed of arterial (A), venous (V), and capillary signals, derived from small vessels within the measurement compartment. The A:V signal ratio fluctuates based on changes in oxygen delivery and oxygen consumption, which enables a reliable trend monitoring of the balance between these two parameters. While the increasing research evidence supports its use, the interpretation of the absolute values of and trends in rcStO2 is still challenging, which halts its routine use in the delivery room and at the bedside. To visualize the influencing factors and improve the understanding of rcStO2 values, we have created a flowchart, which focuses on the three major physiological components that affect rcStO2: oxygen content, circulation, and oxygen extraction. Each of these has its defining parameters, which are discussed in detail in each section.

## Introduction

Near-infrared spectroscopy (NIRS) measurement of the regional cerebral tissue oxygen saturation (rcStO2) in neonates enables continuous non-invasive assessment of oxygen delivery (cDO2) to and oxygen consumption (cVO2) of the frontal brain region. RcStO2 reflects a mixed saturation of the measured microcirculatory compartment, composed of arterial (A), venous (V), and capillary signals (1), whereby the A:V signal ratio fluctuates based on changes in cDO2 and cVO2 within the compartment (2).

NIRS technology relies on the changes in attenuation of light at two or four different wavelengths and their conversion into changes in the concentration of the three chromophores (oxyhemoglobin, deoxyhemoglobin, and cytochrome oxidase) (3). Therefore, NIRS devices measure only relative changes and not absolute concentrations of the chromophores. A new parameter, rcStO2, was introduced in 1999. By using one emission probe and multiple detection probes, the slope of light attenuation vs. distance allowed for the calculation of absolute saturation value (4). Nevertheless, each device on the market has its means of correction for scattering effects and attenuation of the light by superficial tissues, but none of the devices can measure these quantities. It is therefore impossible to express absolute rcStO2 values for an individual patient. Although the measured values of different devices do not display identical numbers, they show a fairly good correlation over time (5), which enables reliable trend monitoring. Normal ranges for the term and preterm neonates at different time points have been established for many of the used devices, respectively (6–10). Furthermore, different manufacturers use different abbreviations for regional cerebral tissue oxygen saturation, as does the published literature. Within this manuscript, we will use the abbreviation rcStO2 to avoid association with any specific manufacturer.

**Figure 1:**
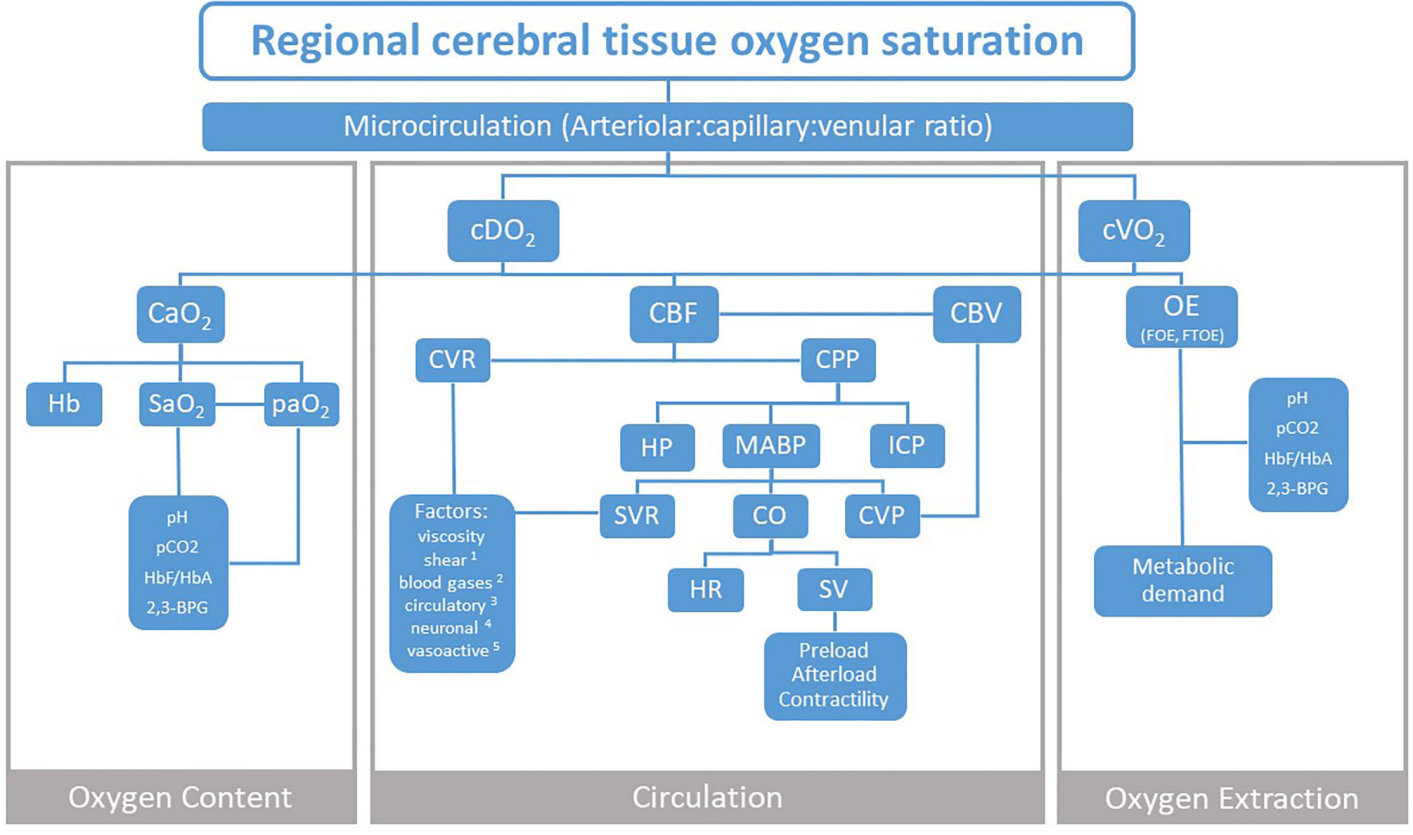
Three physiological components that influence NIRS measurement of regional cerebral tissue oxygen saturation. cDO2, cerebral oxygen delivery; cVO2, cerebral oxygen consumption; CaO2, arterial oxygen content; CBF, cerebral blood flow; CBV, cerebral blood volume; CVR, cerebral vascular resistance; CPP, cerebral perfusion pressure; OE, oxygen extraction; FOE, fractional oxygen extraction; FTOE, fractional tissue oxygen extraction; Hb, hemoglobin; SaO2, arterial oxygen saturation; paO2, arterial partial oxygen pressure; pCO2, partial carbon dioxide pressure; HbF/HbA, fetal to adult hemoglobin ratio; 2,3-BPG, 2,3-biphosphoglycerate; HP, hydrostatic pressure; MABP, mean arterial blood pressure; ICP, intracranial pressure; SVR, systemic vascular resistance; CO, cardiac output; CVP, central venous pressure; HR, heart rate; SV, stroke volume. Factors 1 shear stress and stretch; 2 paCO2, paO2; 3 glucose, adrenaline, ATII- angiotensin II, adenosine; 4 sympathetic, parasympathetic; 5 pH, NO- nitric oxide, ET-1- endothelin-1, EDHF- endothelium-derived hyperpolarizing factor, CNP- C–natriuretic peptide, O2- superoxide.

While the increasing research evidence supports the NIRS measurements, there is still a discussion on its clinical use (11). The interpretation of absolute values of and trends in rcStO2 is still challenging, which halts its routine use at the bedside. For the decision of whether clinical actions should be introduced based on NIRS monitoring, many influencing factors have to be considered. To support those clinical considerations, a comprehensive visualization might be helpful. Therefore, we have created a flowchart, which focuses on the three physiological components that influence rcStO2: Oxygen Content, Circulation, and Oxygen Extraction (OE) (Figure 1).

## Three Physiological Components

### Oxygen Content

#### Arterial Oxygen Content (CaO2)

The most common surrogate for CaO2 is arterial oxygen saturation (SaO2), which can be measured directly in an arterial blood sample by a hemoximeter or continuously non-invasively by pulse oximetry (SpO2). SpO2 reflects the percentage of hemoglobin (Hb) saturated with oxygen, which represents ~99% of total oxygen vs. 1% of oxygen dissolved in the blood. The measured SpO2 is determined by arterial partial oxygen pressure (paO2) and the position of the oxyhemoglobin dissociation curve (ODC) for a given paO2 (12).

In the case of anemic hypoxia, an increase in CaO2 can be achieved by an increase in total Hb (e.g., by transfusion of adult packed red blood cells-RBCs). Multiple studies demonstrated that rcStO2 increases and cerebral OE decreases after RBCs transfusions in anemic preterm neonates. Since transfusion combines several effects (increase in Hb, changes in HbF/HbA ratio and pH, increase in total plasma volume), it is difficult to reliably interpret the most influential parameter (13–16). In addition, there is strong evidence that placental-to-fetal autotransfusion, using delayed cord clamping, increases postnatal rcStO2 (17). This effect combines both an increase in oxygen-carrying capacity (Hb) and an increase in preload, which is a result of the rise in systemic venous return and of the fall in pulmonary resistance (18). In the case of hypoxic hypoxia, improvement of CaO2 can be achieved by an increase in paO2 (e.g., by changing the FiO2 and ventilation settings).

Changes in the ODC position define not only SpO2 values but the OE in the tissues as well. Factors affecting the position of ODC are depicted on the left and right sides of the flowchart (Figure 1).

ODC shift to the left increases Hb oxygen affinity and decreases OE. This can be the result of

1. High fractions of fetal hemoglobin (HbF) in preterm neonates
2. Increase in pH of the blood or decrease in partial carbon dioxide pressure (pCO2) as seen in hyperventilation
3. Lower body temperature (e.g., during therapeutic hypothermia), or
4. Lower concentrations of 2,3-biphosphoglycerate (2,3-BPG) (12, 19).

On the contrary, the shift of the ODC to the right lowers Hb oxygen affinity and increases OE. It is most commonly the result of

1. Adult RBC transfusions with a rapid decline in HbF and rise in adult hemoglobin (HbA) concentration
2. Respiratory acidosis or
3. Increased body temperature (12, 19).

### Circulation

#### Cerebral Blood Flow-CBF

CBF is defined as the blood volume that flows per unit mass per unit time in brain tissue and is typically expressed in ml_blood_/ 100 g_tissue_ min. There are great inter-individual and periodical differences in CBF in human neonates, which makes an establishment of normative values not feasible (20–23).

CBF is regulated on the one hand by cerebral perfusion pressure (CPP) and on the other hand by a cerebrovascular resistance (CVR) or the resistance of the entire cerebral vasculature, whereby small arteries and pial arterioles, which can regulate their radius through vasodilatation and vasoconstriction, account for most of the CVR changes (24). CVR is determined by vascular smooth muscle tone, which is under the influence of neural, chemical, metabolic, and physical factors (25) (Figure 1).

#### Cerebral Vascular Resistance-CVR

The two most important parameters that regulate CVR are changes in the partial pressure of carbon dioxide (paCO2) and, to a lesser degree, in the partial pressure of oxygen in arterial blood (paO2) (26). Higher paCO2 (hypercapnia) leads to vasodilatation (lower CVR) and an increase in CBF, whereas lower paCO2 (hypocapnia) generates vasoconstriction (higher CVR) and a decrease in CBF (27–29). Lower CaO2 during hypoxia, on the other hand, has a similar effect as hypercapnia and causes a dilatation of pial vessels which increases CBF (30). Furthermore, it is important to note that the changes in paO2 and paCO2 influence both ventilation rate and CBF, whereby these vary inversely in response to paCO2 and paO2 levels. Hypoxia and hypercapnia induce the activation of peripheral chemoreceptors, which leads to hyperventilation, reduction in pCO2, and cerebral vasoconstriction (25). The final effect on CVR depends on the paO2 to paCO2 ratio. A low paO2 to paCO2 ratio results in a greater degree of hypoxic vasodilatation for a given hypocapnic vasoconstriction (31). The affection of CBF by hyperoxia (paO2 > 15 kPa/113 mmHg) was studied in preterm and term infants and a reduction in CBF as well as a reduced vasoreactivity in preterm compared to term neonates could be demonstrated (32, 33).

The effect of paCO2 is mediated through changes in pH (i.e., H+ concentrations). Hypercapnia leads to an increase in H+ concentration in the perivascular space, which increases K+ outflow from smooth muscle cells of cerebral arteries and arterioles and causes muscular relaxation, i.e., vasodilatation (34). The molecular mechanism which regulates the paO2 effect on CBF is quite different and it requires an intact endothelium and nitric oxide (NO) production. Newer studies suggest that deoxyhemoglobin (associated with changes in CaO2), rather than paO2, serves as the primary biological regulator of CBF and induces the release of NO metabolites and adenosine triphosphate with consequential vasodilatation (35). However, hypoxia can also induce tissue lactacidosis, and the resulting increase in H+ concentrations provides a link between CO2- and O2-mediated regulation of CVR and CBF (21). In addition, CBF is also influenced, in the long term, by other hypoxia-induced changes (e.g., increased capillary density using angiogenesis, increased hematocrit, and viscosity) (25).

CBF varies inversely with hematocrit in many species in both acute (i.e., acute anemia) and chronic conditions (i.e., erythropoiesis). There are two possible mechanisms involved both resulting in changes in CVR. In acute anemia, a decrease in CaO2 induces a cerebral vasodilatory response to maintain a constant cDO2 (36). In conditions of hyperviscosity, shear stress/stretch mechanisms induce adaptations of CVR to maintain a constant CBF (37).

Another parameter that influences CBF is blood glucose. In preterm neonates, a compensatory increase of CBF during uncomplicated hypoglycemia could be demonstrated (38, 39). The described mechanism behind it could be the capillary recruitment, which can be measured by NIRS as an increase in the cerebral blood volume (CBV) during hypoglycemia and a decrease in CBV after intravenous glucose infusion (40).

Other factors play a minor role in the regulation of CVR and affection of CBF and are therefore only listed in the flowchart, but not discussed in detail (Figure 1).

#### Cardiocirculatory Parameters

The mean arterial blood pressure (MABP) is influenced by the volume state that determines central venous pressure—CVP, by systemic vascular resistance (SVR) of the arterial tree, and by cardiac output (CO), which is a product of heart rate (HR) and stroke volume (SV).

In a healthy neonate, CO is mostly determined by the metabolic rate of the peripheral tissues (41). The adaptation of CO to meet the metabolic demands includes changes in SV and HR. Although there is a common belief that neonates cannot alter SV and that tachycardia is the primary mean of increasing CO, several studies have demonstrated the opposite. According to studies performed in healthy term neonates during and after transition, SV and not HR was the main determinant of neonatal left ventricular output (42–44). This can be explained by a postnatal increase in the left ventricular preload in combination with ductal left-to-right shunting, due to a decrease in pulmonary vascular resistance. Furthermore, a study, which established normative values for non-invasive measurement of CO using electrical cardiometry, demonstrated an increase in SV and CO with increasing birth weight and gestational age. The best model to describe the relationship between SV, CO, and birth weight or gestational age was exponential (45). Another study conducted in healthy term neonates during the first postnatal days found that SV and CO decrease in response to a short duration of prone positioning, while HR, SpO2, and rcStO2 do not change significantly (46). In preterm neonates who have a high resting HR and an intrinsic diastolic dysfunction, an increase in CO is also determined mainly by changes in SV (47, 48). Moreover, excess tachycardia (due to pain or agitation and caffeine citrate administration) as well as bradycardia (due to sleep apnea and immaturity) can both reduce CO in preterm neonates.

SV is determined by preload, afterload, and myocardial contractility. Preload represents the initial stretching of the cardiac muscle fibers before contraction (mostly referred to as an end-diastolic ventricular volume), whereas afterload can be best described as the force resisting the ejection of blood by the heart (49).

Preload depends on venous return, which is determined by the circulating volume, venous capacitance, and ventricular compliance. The fetal myocardium contains specific isoforms of fetal connection which renders its higher compliance compared to the adult heart (50). This allows the fetal heart to generate adequate CO despite low-filling pressures *in utero*. In addition, an early increase in preload after delayed cord clamping increases CO during transitional circulation and results in higher rcStO2 at 4 and 24 h after birth in preterm neonates (17, 18).

The afterload largely depends on ventricular dimensions, MABP, SVR, and vascular compliance. Echocardiographic studies showed that the neonatal heart has a higher basal contractile state and that myocardial performance is more sensitive to afterload in the immature heart (51, 52). Therefore, a rapid increase in SVR due to clamping of the umbilical cord at the beginning of fetal to neonatal transition results in a reduced CO immediately after birth. These changes can lead to cerebral hypoperfusion even if MABP remains in the perceived normal range (53, 54).

Regulation of SV by an increase in contractility is limited in fetuses and neonates due to myocardial immaturity (55). The increase in contractile force (secondary to an increase in calcium influx) is age-dependent and improves during the early postnatal period (49).

Many studies in the adults have found a positive relationship between CO and CBF (56, 57). The left CO is positively correlated to CBF in neonates as well (58). An impaired cardiac function can, therefore, result in CBF reduction, and consequentially, in decreased cDO2.

#### Cerebral Blood Volume-CBV

CBV can be derived from a NIRS measurement as ΔCBV and expressed in ml_blood_ */*100 g_braintissue_ if the changes in the total Hb during the measurement and Hb concentration from a large vessel are known (59). With the time-resolved NIRS devices, it is possible to measure the absolute value of CBV (60). CBV is sensitive to changes in paO2 and paCO2, similarly to CBF (59, 61). An increase in CBV after functional obstruction of the homolateral jugular vein has also been reported (62, 63). CBV is thus related to CBF on the arterial side, and to CVP on the venous side and can be used as a surrogate for the assessment of cerebral hemodynamics in neonates.

#### Cerebral Autoregulation

Under stable conditions, CBF is maintained over a wide range of MABP as a result of cerebral autoregulation. The driving pressure of CBF is CPP or the difference between MABP and intracranial pressure (ICP), i.e., the pressure of the cerebrospinal fluid in the subarachnoid space (24).

CBF is coupled to cerebral oxygen metabolism to ensure appropriate cDO2 at baseline and in response to cortical activity (64). This metabolic coupling mechanism is one type of cerebral autoregulation. Another type of cerebral autoregulation enables cerebral arteriolar caliber to adjust and ensure stable CBF relatively independently of changes in MABP (65). This phenomenon can be illustrated by a flat sigmoidal curve with stable CBF over a wide range of tolerable MABP and impairment at either extreme (66).

The CBF independency of MABP was described in several studies in clinically stable mechanically ventilated neonates (67), where constant cerebral perfusion was maintained in MABP ranges of 25–40 mmHg (68). Other works, however, described a linear relationship between CBF and MABP in ill neonates, independently of the severity of their postnatal condition (23, 69, 70).

The terms pressure-passive circulation and impaired autoregulation were introduced to describe the failure of the preterm cerebral vasculature to maintain uniform CBF over a range of MABP. The frequency of impaired autoregulation is associated with low gestational age and birth weight as well as with systemic hypotension. An impaired autoregulation results in unstable CBF, generating the cycle of ischemia–reperfusion, which is the main mechanism of preterm brain injury (66). There is also a direct link between increased periods of impaired autoregulation and adverse neurological outcome such as intracranial hemorrhage (71).

### Oxygen Extraction-OE

#### OE Depends on a Tissue Metabolic Activity

OE is the amount of oxygen removed from the arterial circulation by tissue within a certain period. It can be best defined as a difference between CaO2 and CvO2 (venous oxygen content). The extraction of oxygen from a hemoglobin molecule to a tissue depends on the difference between paO2 in the vessels and local partial oxygen pressure in the tissue and is hence determined by the position of ODC. The amount of extracted oxygen depends mainly on tissue metabolic activity in a state of sufficient cDO2 (64). The CaO2 range, over which a normal OE can be sustained, varies from one tissue group to another, depending on their metabolic activity and priority (vital vs. non-vital organs) (65). The tissue metabolic activity differs considerably also within organ systems, depending on factors such as circadian rhythm (heart, endocrine tissue), feeding (gastrointestinal system), thermoregulation (fat tissue), and muscular activity (heart, lungs, muscles). NIRS studies mostly report cerebral OE in terms of cerebral fractional tissue OE (cFTOE), which represents the difference between SpO2 and rcStO2 (mixed saturation). Some studies preform the calculation of cerebral fractional OE (cFOE), which represents the difference between the arterial and venous compartment and its measurement requires partial jugular venous occlusion (72).

### Microcirculation

As already mentioned above, rcStO2 reflects a mixed saturation derived from small vessels (<0.1 mm in diameter) within the measurement compartment. The A:V signal ratio fluctuates based on changes in cDO2 and cVO2 within this microcirculatory compartment (2). As long as cerebral autoregulation is within limits, resting global cDO2 can be considered as a constant variable and cVO2 changes in response to brain metabolic demand. Although the A:V contribution to rcStO2 is commonly rounded to the 25:75 ratio, the largest *in vivo* study conducted in neonates and young children showed an average, relatively constant ratio of 15:85 in different experimental conditions (normoxia, hypoxia, and hypocapnia) (73). Nonetheless, the A:V ratio differed significantly among individual patients and was unaffected by their demographic and physiological characteristics. There seems to be a biological variation in the A:V ratio, which does not change substantially during hypoxia or hypocapnia (73). One has to be aware of this important fact when interpreting rcStO2 values in an individual patient.

## Discussion

In this article, we schematically present the three physiological components that influence the measurement of rcStO2. An important aim was to improve the understanding of the relationships between the parameters, in order to interpret rcStO2 easily in the clinical routine. Very recently, a paper focusing on critical appraisal of methods used for the assessment of cerebral oxygenation has been published (11). The authors presented a mechanistic model of variables affecting the local tissue partial oxygen pressure. This model partially resembles our flowchart, since it depicts the same parameters that affect CO, MABP, SaO2, and end-capillary-hemoglobin-oxygen saturation. However, the focus of the paper was not on describing the physiological components, but rather on the discussion of the benefits and risks involved in using electric cardiometry, invasive blood pressure measurements, pulse oximetry, and cerebral NIRS to assess the latter. We think that both presentations may complement each other very well.

Numerous studies showed the affection of rcStO2 by different parameters of our flowchart. Regarding those which focused on immediate fetal to neonatal transition, the findings are rather specific and not always as per the later periods. For instance, rcStO2 and SpO2 were positively correlated in several studies in preterm neonates, as expected (74–76). Higher pCO2 was, however, unexpectedly associated with lower rcStO2 in preterm in contrast to no associations in term neonates, suggesting a less pronounced vasodilatory effect of pCO2 in preterm neonates during the transition (77). Higher blood glucose concentrations were, as expected, associated with lower rcStO2 in both preterm and term neonates (78). Furthermore, no significant correlations between rcStO2 and MABP in preterm and term neonates were described (79). Interestingly, in term neonates with uncomplicated neonatal transition after Cesarean section, rcStO2 did not correlate with CO (80).

Concerning cerebral perfusion, it is important to keep in mind that CO cannot be used as a direct surrogate for systemic or cerebral blood flow in neonates because of a high incidence of shunts through the ductus arteriosus and atrial septum. However, flow measurements in the superior vena cava (SVC) can assess blood returning from the upper body and brain (81). The following two studies were conducted in VLBW neonates during the first days after birth and without detectable brain pathology: (i) the first study described a positive correlation between rcStO2 and SVC flow, but a poor correlation between rcStO2 and CO during the first postnatal day (82); (ii) the second study, however, reported a negative correlation between rcStO2 and SVC flow at 6 h after birth and no relevant correlations of rcStO2 with either SVC flow or CO during the following 48 h after birth. Both the SVC flow and CO increased during this period, but rcStO2 decreased at 12 h of age.

Although the evidence of a direct correlation between rcStO2 and CO in stable neonates is lacking, an important finding is a positive correlation between rcStO2 and CO in neonates who develop IVH at 24 h after birth (83). Namely, lower rcStO2 as well as CBF passivity to systemic blood flow, reflected in the correlation between rcStO2 and MABP or CO have been predictors of several neurological adverse effects, such as intraventricular and periventricular hemorrhage (IVH, PVH) and periventricular leukomalacia (PVL) (84–87). These correlations, both positive and negative, indicate an impaired cerebral autoregulation in preterm neonates (88, 89). Moreover, in term neonates with hypoxic-ischemic encephalopathy treated with therapeutic hypothermia, rcStO2 and CBF correlate predominantly with right ventricular function (90). These findings further stress the complexity of CBF regulation in both preterm and term neonates. It can be concluded that CBF can be independent of MABP and CO and that this indicates intact cerebral autoregulation. This can also explain how even very low CBF can be consistent with healthy survival.

For MABP independent CBF, changes in CPP are either a result of the changes in HP or ICP. Several NIRS studies have described significant changes in NIRS-derived CBV measurements as a result of changes in HP after head tilting in healthy and in preterm neonates with PVL (91, 92). However, rcStO2 was not affected or changed only minimally during the maneuver (93, 94). The affection of rcStO2 by the changes in ICP was examined in very few studies. Preterm neonates with hydrocephalus accompanied by intracranial hypertension had significantly lower rcStO2 in a study that compared invasive ICP measurements with NIRS parameters (95). Ventricular drainage with ICP reduction after post-hemorrhagic hydrocephalus led to an increase in rcStO2 or to an improvement in CBF in several studies (96–98).

Regarding rcStO2 and the pulse-oximetry parameters, rcStO2 was positively correlated to SpO2 and HR during the first 72 h in healthy preterm neonates (99, 100). However, a lower percentage of significant cross-correlations were observed in patients with IVH or PVH compared to healthy controls (99). As for the associations with the parameters of CaO2 or cVO2 depicted on the left and the right of our algorithm, there is evidence of higher rcStO2 at 24 h after birth in association with delayed cord clamping and higher hematocrit or Hb in preterm neonates (17). On the contrary, elevated OE is reported as an adaptation to anemia of prematurity (101). Additionally, RBC transfusions improve rcStO2 and lead to a decrease in cFTOE in anemic preterm neonates (13–16). There is still no evidence that the ODC shift to the right is related to lower fractions of HbF after the RBC transfusions, and results in higher rcStO2, as physiologically expected, but there are also very few studies currently available (102).

Although there are many benefits of rcStO2 monitoring, a clinician has to be aware of the limitations of NIRS technology. RcStO2 monitoring has to be continuous to have an impact on clinical outcomes. However, the repeated measurements within the subject showed a standard deviation of 5 to 6% (103), and a systematic bias between sensors from INVOS 5100 and NIRO 300 was reported (104). Furthermore, although most NIRS devices estimate the rcStO2 every 5 s, the clinical interpretation should be based on the trend over minutes and hours. Since the first week after birth is the most vulnerable period concerning low CBF and high risk of adverse outcomes, NIRS monitoring may be initiated immediately or within hours after birth to be most efficient (105). Although it is a very safe method, local skin irritation, sores, and redness have been reported as adverse effects of NIRS devices (106).

Nevertheless, the clinical value of continuous NIRS monitoring is still under discussion (11). Two randomized interventional studies were undertaken to investigate possible short- and long-term benefits. The interventional studies instead of two interventional studies defined rcStO2 ranges, either during immediate transition and resuscitation after birth (COSGOD) or during the first 72 h after birth (SafeBoosC). Both phase II studies reported short-time benefits regarding the cerebral burden of hypoxia, but evidence for long-term benefits is still lacking. The SafeBoosC II trial, which aimed to keep rcStO2 within the 55–85% range (107), demonstrated that the evidence-based treatment guideline vs. a blinded collection of rcStO2 and treatment as usual, significantly reduced the burden of cerebral hypoxia (108) without affecting the electroencephalographic (EEG) outcomes, blood biomarkers of cerebral injury (109), or the incidence of severe cerebral injury as assessed with imaging methods (110). Moreover, a follow-up neurodevelopmental study at 2 years of corrected age showed no long-term benefits or harm for the experimental group (111). The trial also showed a higher prevalence of bronchopulmonary dysplasia and retinopathy of prematurity in the experimental group (112). Similarly, the COSGOD II trial could demonstrate a reduction of the burden of cerebral hypoxia during immediate transition and resuscitation after birth, whereas the cerebral injury rate and neurologic outcome at term age were not different between the experimental and control group (74). The results of the SafeBoosC III trial, with the primary objective to decrease a composite of either death or severe brain injury detected on any of the serial cranial ultrasound scans in preterm neonates <28 weeks' gestation and COSGOD III trial, with the primary objective to increase survival without cerebral injury in preterm neonates <32 weeks' gestation, are still awaited (112, 113).

In summary, continuous rcStO2 monitoring in human neonates enables a reliable trend monitoring of cerebral oxygen metabolism and early recognition of compromised cerebral oxygen delivery and consumption. The interpretations of changes of rcStO2 may be a challenge for clinicians, as it is difficult to be aware of all the potential influencing factors. Nevertheless, predefined actions directed to respiratory and cardiovascular stabilization in case of low rcStO2 values were able to reduce the cerebral burden of hypoxia in randomized intervention studies. The present concept of three influencing compartments may be helpful to guide clinical actions in association with the use of NIRS monitoring.

## Data Availability Statement

The original contributions presented in the study are included in the article/supplementary material, further inquiries can be directed to the corresponding authors.

## Author Contributions

BU and GP conceptualized and designed the flowchart. ES conducted a search of the literature, drafted the initial manuscript, and reviewed and edited the manuscript. CB-H and BS designed the flowchart and reviewed and edited the manuscript. ES, GP, CB-H, BS, and BU critically reviewed the manuscript for important intellectual content. All authors approved the final manuscript as submitted and agree to be accountable for the content of the work.

## Conflict of Interest

The authors declare that the research was conducted in the absence of any commercial or financial relationships that could be construed as a potential conflict of interest.

## Publisher's Note

All claims expressed in this article are solely those of the authors and do not necessarily represent those of their affiliated organizations, or those of the publisher, the editors and the reviewers. Any product that may be evaluated in this article, or claim that may be made by its manufacturer, is not guaranteed or endorsed by the publisher.
